# Enhanced RHO‐ROCK signaling is associated with CRELD2 production and fibroblast recruitment in cutaneous squamous cell carcinoma

**DOI:** 10.1002/cm.21894

**Published:** 2024-07-09

**Authors:** Alexandra Pittar, Edward J. Buckley, Sarah T. Boyle, S. Jan Ibbetson, Michael S. Samuel

**Affiliations:** ^1^ Centre for Cancer Biology an Alliance between SA Pathology and the University of South Australia Adelaide Australia; ^2^ Division of Surgical Pathology SA Pathology Adelaide Australia; ^3^ Adelaide Medical School, Faculty of Health and Medical Sciences University of Adelaide Adelaide Australia; ^4^ Basil Hetzel Institute for Translational Health Research Woodville South Adelaide Australia

**Keywords:** cancer‐associated fibroblasts, CRELD2, cutaneous squamous cell carcinoma, extracellular matrix, RHO‐ROCK signaling

## Abstract

A key characteristic of cancer cells is their ability to induce changes in their microenvironment that render it permissive to tumor growth, invasion and metastasis. Indeed, these changes are required for tumor progression. Consequently, the tumor microenvironment is emerging as a key source of new targets against cancer, with novel therapies aimed at reversing tumor‐promoting changes, reinstating a tumor‐hostile microenvironment and suppressing disease progression. RHO‐ROCK signaling, and consequent tension within the cellular actomyosin cytoskeleton, regulates a paracrine signaling cascade that establishes a tumor‐promoting microenvironment. Here, we show that consistent with our observations in breast cancer, enhanced ROCK activity and consequent production of CRELD2 is associated with the recruitment and tumor‐promoting polarization of cancer‐associated fibroblasts in cutaneous squamous cell carcinoma. Our observations provide support for the notion that the role of RHO‐ROCK signaling in establishing a tumor‐promoting microenvironment may be conserved across patients and potentially also different cancer types.

## 1 INTRODUCTION

Cutaneous squamous cell carcinomas (cSCC) are amongst the most frequently diagnosed cancers, usually arising from cumulative sun damage. While detailed records of cSCC incidence are generally not maintained by most countries, the most recent estimates suggest 200,000–400,000 new diagnoses and 3000 deaths are attributable each year to this form of skin cancer in the USA, with incidence rising (Kim et al., 2018). Whereas early lesions may be easily treated by excision, untreated or incompletely excised cSCCs pose a significant risk of invasion and metastasis. Uncovering novel biomarkers that may predict the development of invasive and metastatic disease would therefore be useful in informing how patients are managed following excision of the primary lesion.

The invasive and metastatic cSCC phenotypes are underpinned by changes in the tumor microenvironment (TME), including the appearance of cancer‐associated fibroblasts (CAFs) (Sahai et al., 2020), a dense extracellular matrix (ECM) and tumor‐associated inflammation. It is now well appreciated that the diverse population of CAFs have key roles in regulating cancer cell proliferation, immune cell recruitment/infiltration and specific phenotypes exhibited by these cell types either directly, or indirectly via their role in establishing and maintaining the tumor ECM (Kolesnikoff et al., 2022). Consequently, the CAF population is fundamental to cancer progression, promoting cancer cell invasion and metastasis and thereby determining outcomes for patients.

An outstanding question in the field has been the nature of !mechanisms linking epidermal cell transformation to changes in CAF phenotype that regulate cSCC progression. We have previously demonstrated that signaling through the RHO‐ROCK pathway, which regulates structural and functional characteristics of the cellular actomyosin cytoskeleton, promotes tumor progression via cancer‐promoting changes in the microenvironment (Samuel, Lopez, et al., 2011). Crucially, these changes are dependent on the role of RHO‐ROCK signaling in enhancing actomyosin contractility, and ROCK kinase activity is causally related to enhanced growth and invasiveness in cSCC (Ibbetson et al., 2013; Samuel, Lopez, et al., 2011). RHO‐ROCK pathway activation in cancer cells within the cSCC tumor mass further drives changes in the phenotype of tumor‐associated fibroblasts, and the consequent enhancement of collagen production, which stiffened the ECM and activated mechanotransduction signaling pathways within tumor cells, that promoted disease progression (Kular et al., 2015).

Using a parallel approach in murine mammary cancers, we found that ROCK activation in cancer cells induces the production and secretion by these cells of Cysteine‐rich with EGF‐like domains 2 (Creld2), which acts on tumor fibroblasts, converting them to a tumor‐promoting form of CAFs and promoting mammary tumor progression (Boyle et al., 2020). Furthermore, high levels of CRELD2 in breast cancers were associated with reduced overall survival, invasiveness and triple‐negative status (in which the loss of the estrogen receptor, progesterone receptor and the human epidermal growth factor receptor 2 results in a highly aggressive form of cancer), in human breast cancer patients (Boyle et al., 2020). Given that RHO‐ROCK signaling promotes tumor progression in cSCC, we therefore wondered whether CRELD2 has a role in cSCCs exhibiting ROCK activation and if so, whether this was associated with enhanced fibroblast infiltration in human patients. To answer this question, we took advantage of a collection of cSCC specimens that we had previously characterized for ROCK activity and ECM changes (Ibbetson et al., 2013), and investigated whether there was an association between CRELD2 production and changes in the CAF population, with a particular focus on invasive regions relative to hyperplastic, non‐invasive regions of the same tumors compared to margin skin. Our findings suggest that CRELD2 levels correlate strongly with CAF phenotype, with the potential to impact on overall patient survival.

## 2 RESULTS

### 2.1 RHO‐ROCK signaling is elevated in cSCC

**FIGURE 1:**
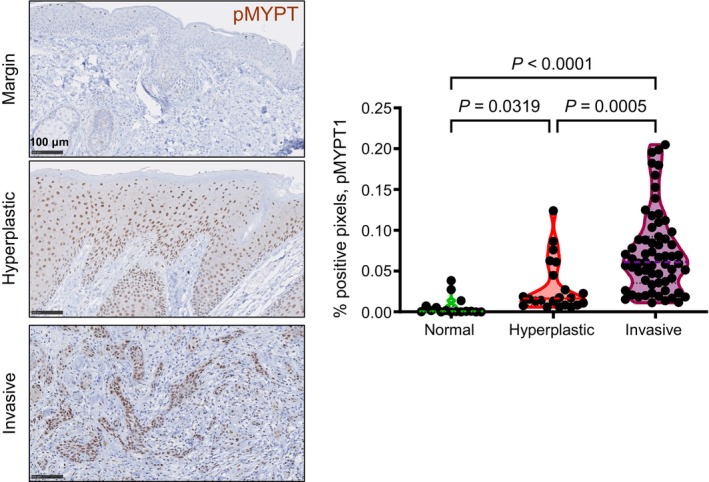
ROCK is progressively activated in cutaneous squamous cell carcinoma. Representative images of immunohistochemical staining (brown) for pThr696‐MYPT1, in margin skin, hyperplastic skin tissue and invasive cSCC. Sections have been counterstained with hematoxylin. Violin plots show data from positive pixel analysis of images derived from sections stained for pMYPT1. Medians (color dashed lines) and inter‐quartile ranges (black dashed lines) are indicated. Statistical significance was derived using the Kruskal–Wallis test.

ROCK is a Ser‐Thr kinase that regulates the activity of a suite of substrate proteins that govern cytoskeletal structure and function (Amano et al., 2010). Consequently, it is involved in an array of cellular processes that require changes to the cytoskeleton, including cell division, migration, membrane dynamics and apoptosis. Given the role of these processes in cancer growth and progression, it has been demonstrated that ROCK is activated and that this is required for disease progression in a variety of different cancers, including those of the skin (Samuel, Lopez, et al., 2011; Sanz‐Moreno et al., 2011); breast (Boyle et al., 2020), and pancreas (Rath et al., 2017). Crucially, ROCK activity, as indicated by the phosphorylation status of the ROCK substrate myosin phosphatase targeting subunit I at Thr‐696 (Feng et al., 1999), strongly correlates with histological characteristics, with activity progressively increased from margin skin to hyperplastic epidermis, with the highest activities observed in invasive regions of cSCCs (Figure 1). This is consistent with previous observations that signaling through ROCK is progressively activated in cSCC, and that this is causally linked with enhanced production of the ECM proteins, collagen, fibronectin, periostin and tenascin C within the microenvironment (Ibbetson et al., 2013). As an aside, inactive pMYPT1 is observed to accumulate within the nucleus, consistent with previous reports (Wu et al., 2005), one hypothesis is that this may serve to physically separate MYPT1 from substrates located principally within the cytoplasm, although this remains to be tested.

### 2.2 Fibroblast recruitment is progressively enhanced in human cutaneous squamous cell carcinoma

**FIGURE 2:**
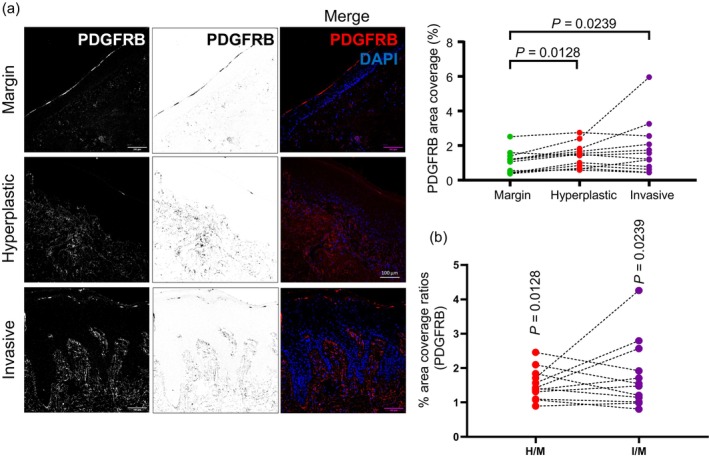
Fibroblast infiltration is enhanced in cutaneous squamous cell carcinoma. (a) Representative images of immunofluorescence analysis (white in dark‐field and grayscale in bright‐field monochromatic images, and red in merged images) for PDGFR‐betB, in margin skin, hyperplastic skin tissue and invasive cSCC. Sections have been stained with DAPI to label nuclei. Line plots show data from integrated density analysis of images derived from sections labelled for PDGFRB. Each dot represents a single tumor. Margin, hyperplastic and invasive regions from the same tumor are linked by a dotted line. Statistical significance was derived using the Friedman test. (b) Chart shows ratios of PDGFRB area coverage relative to margin, for each tumor. Statistical significance comparing each ratio to 1, was derived using the Friedman test.

In breast cancers, ROCK activity results in the enhanced infiltration of fibroblasts into the microenvironment, resulting from paracrine signals emanating from cancer cells. Indeed, it is these fibroblasts that are the major source of ECM proteins that are enriched in cSCCs relative to margin tissue (Boyle et al., 2020). In order to ascertain whether cSCCs also exhibit this phenomenon, we undertook immunofluorescence analysis for PDGRFB, which is widely used as a general marker of fibroblasts (Nurmik et al., 2020). Our analysis of a cohort of primary SCC specimens in which we had previously characterized ROCK pathway activation and ECM protein production (Ibbetson et al., 2013) revealed that fibroblast infiltration is enhanced in both hyperplastic skin tissue and invasive cSCC regions compared to margin skin (Figure 2), and this is consistent with previous reports that ROCK activity enhances the production of ECM proteins in cSCC (Ibbetson et al., 2013), and is similar to the observation in breast cancers, that ROCK activity causes the enhanced recruitment of fibroblasts that produce a tumor‐promoting ECM (Boyle et al., 2020).

### 2.3 CRELD2 production is enhanced in human cutaneous squamous cell carcinoma

**FIGURE 3:**
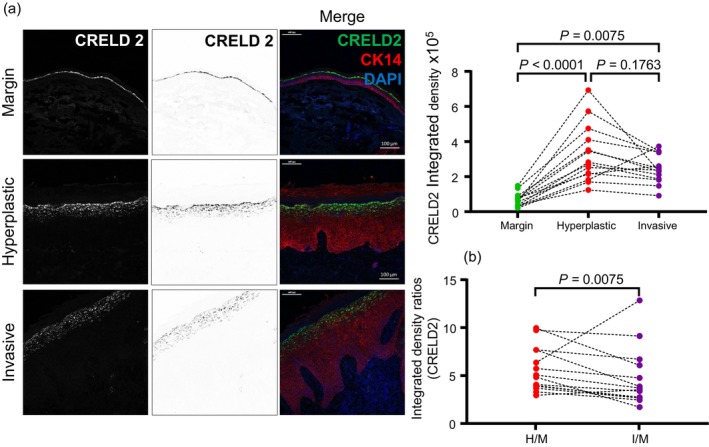
CRELD2 is elevated in cutaneous squamous cell carcinoma. (a) Representative images of immunofluorescence analysis (white in dark‐field and grayscale in bright‐field monochromatic images and green in merged images) for CRELD2, in margin skin, hyperplastic skin tissue and invasive cSCC. Sections have been stained with DAPI to label nuclei and an antibody against cytokeratin 14 (CK14) to label epidermis. Line plots show data from integrated density analysis of images derived from sections labelled for CRELD2. Each dot represents a single tumor. Margin, hyperplastic and invasive regions from the same tumor are linked by a dotted line. Statistical significance was derived using the Friedman test. (b) Chart shows ratios of CRELD2 integrated densities relative to margin, for each tumor. Statistical significance was derived using the Wilcoxon test.

Since fibroblast recruitment is progressively increased in cSCC, we wondered whether CRELD2, a key paracrine factor involved in the recruitment and polarization of tumor‐promoting CAFs in autochthonous mammary tumors (Boyle et al., 2020), may also be involved in regulating the enhanced infiltration of fibroblasts into cSCCs. We investigated CRELD2 levels in the cohort of cSCC specimens using a validated antibody (Figure 3). Margin skin, which had been assessed histo‐pathologically to contain no cancer cells, exhibited relatively low levels of CRELD2, and hyperplastic regions exhibited higher levels. On the other hand, while CRELD2 is elevated in invasive regions compared to margin skin, invasive regions of cSCC do not appear to express higher CRELD2 levels than hyperplastic regions—and CRELD2 levels may in fact be a little lower in invasive regions compared to hyperplastic regions. However, it is difficult to be definitive on this point as the differences in CRELD2 levels between hyperplastic and invasive regions were not statistically significant. Nevertheless, these data are consistent with the notion that CRELD2 upregulation in cSCCs may be involved in the recruitment of fibroblasts into the microenvironment as previously demonstrated in the case of breast cancers (Boyle et al., 2020). Interestingly, CRELD2 appears to be principally produced by squamous epithelium (Figure 3), which is consistent with our observation that CRELD2 is secreted by mammary tumor cells of epithelial origin in breast cancer (Boyle et al., 2020).

### 2.4 Tumor‐promoting CAFs are enhanced in human cutaneous squamous cell carcinoma

**FIGURE 4:**
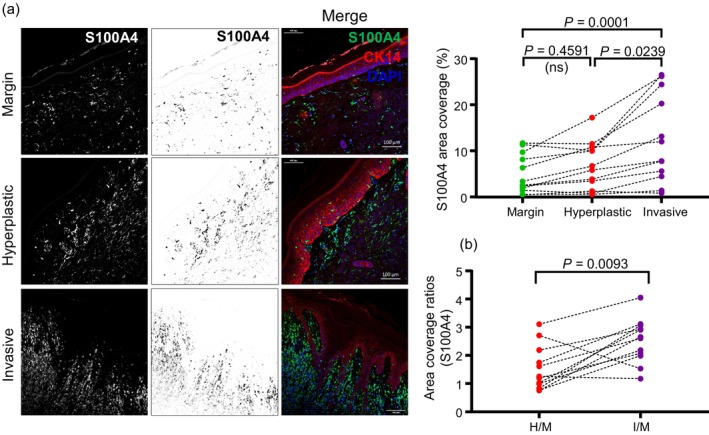
Tumor‐promoting CAFs are enriched in cutaneous squamous cell carcinoma. (a) Representative images of immunofluorescence analysis (white in dark‐field and grayscale in bright‐field monochromatic images and green in merged images) for CAF marker S100A4, in margin skin, hyperplastic skin tissue and invasive cSCC. Sections have been stained with DAPI to label nuclei and an antibody against cytokeratin 14 (CK14) to label epidermis. Line plots show data from integrated density analysis of images derived from sections labelled for S100A4. Each dot represents a single tumor. Margin, hyperplastic and invasive regions from the same tumor are linked by a dotted line. Statistical significance was derived using the Friedman test. (b) Chart shows ratios of S100A4 area coverage relative to margin, for each tumor. Statistical significance was derived using the Wilcoxon test.

We next wondered whether the fibroblasts recruited into the microenvironment of cSCCs exhibited markers associated with tumor promotion. The S100 calcium‐binding protein family member S100A4 is upregulated in tumor‐promoting CAFs (Nurmik et al., 2020) and frequently used as a marker to identify this population of CAFs. We therefore conducted immunofluorescence analysis of S100A4 on the cohort of cSCCs we had investigated for ROCK activation, PDGFRB+ fibroblast infiltration and CRELD2 production above. These analyses revealed that margin skin exhibited low S100A4+ CAF infiltration, while invasive regions of cSCC exhibited the greatest S100A4+ CAF infiltration (Figure 4). While hyperplastic skin exhibited some S100A4+ CAFs, these were comparable to the levels observed in margin skin. This timing is consistent with our previous observations that CRELD2 production caused the recruitment of fibroblasts (Boyle et al., 2020).

### 2.5 CRELD2 levels strongly correlate with fibroblast infiltration into human cutaneous squamous cell carcinoma tissue and may influence survival

**FIGURE 5:**
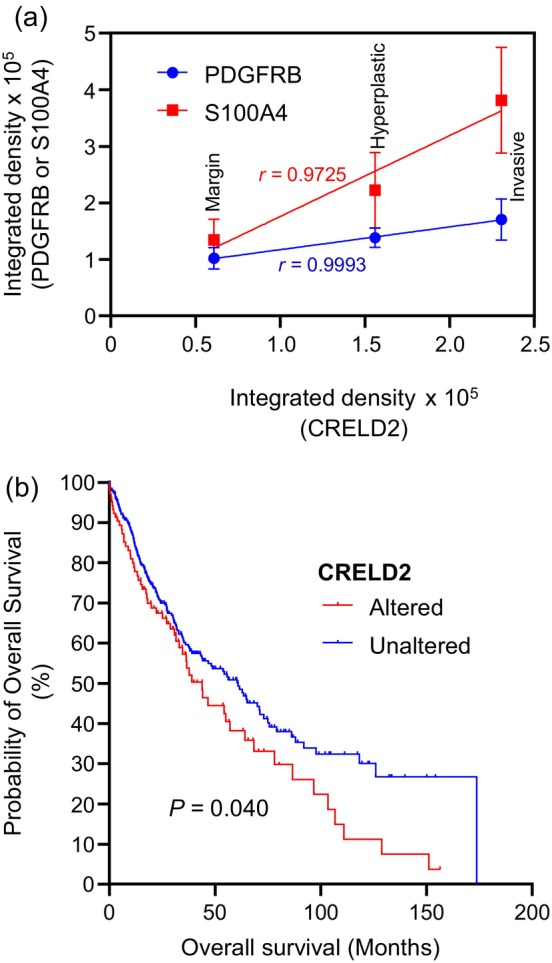
CRELD2 strongly correlates with fibroblast infiltration and reduced patient survival. (a) Graph shows mean integrated density values for CRELD2 immunofluorescence plotted against integrated density values for PDGFRB and S100A4 immunofluorescence across margin, hyperplastic and invasive cSCC tissue. Error bars represent standard error of the mean. Pearson *r* values derived from simple linear regression analyses are also shown. (b) Survival data for lung squamous cell carcinoma expressing altered CRELD2 levels versus control.

Since we observed strong associations between CRELD2, PDGFRB and S100A4, we wondered whether there may be a mathematical correlation between these factors. To this end, we investigated whether CRELD2 levels as measured by integrated density of immunofluorescence labeling—a measure of fluorescence intensity—correlated separately with those of PDGFRB or S100A4. Plotting the median values of these intensities for margin skin, hyperplastic tissue and invasive cSCC revealed a significant linear correlation (Pearson *r* value of .9993) between CRELD2 levels and those of PDGFRB (Figure 5a). These observations strongly suggest that, as in breast cancer, CRELD2 may also regulate the recruitment of fibroblasts in cSCC. Furthermore, we also observed a strong correlation between CRELD2 levels and those of the CAF marker S100A4, suggesting that CRELD2 may also be involved in establishing a tumor‐promoting CAF phenotype in cSCC. Interestingly, this observation also strongly mirrors our findings in breast cancer (Boyle et al., 2020).

We next wondered whether CRELD2 levels in SCC could be informative for patient survival. Given the lack of gene expression data in cSCC, we queried cBioPortal for squamous cell carcinomas of other tissue origins for which mRNA expression data had been reported. Typical of our observations is that presented in Figure 5b, where overall survival was lower in patients whose lung squamous cell carcinomas expressed altered levels of CRELD2, with a median survival of 43.9 months relative to those patients expressing unaltered CRELD2 levels, who had a median survival of 61 months. Taken together, these data suggest that CRELD2 may regulate the recruitment and tumor‐promoting polarization of CAFs and may therefore impact patient survival in squamous cell carcinoma.

## 3 DISCUSSION

Regulation of the cellular cytoskeleton is fundamental to myriad cellular processes, arising from the strong link between cellular structure and function. Accordingly, signaling through the RHO‐ROCK pathway, which is strongly upregulated in various cancers, influences not only cell autonomous functions, but also acts non‐cell autonomously to regulate both the tissue and TME (Boyle et al., 2020; Kular et al., 2015; Samuel, Lopez, et al., 2011). One way in which RHO‐ROCK signaling regulates the TME is via the production and secretion of CRELD2, an enigmatic protein that is known to also be secreted under conditions of ER stress (Tang et al., 2023). We have previously demonstrated that CRELD2 secretion enhances the tumor‐promoting characteristics of CAFs in breast cancer. Here we have adopted a correlative approach to demonstrate in patient samples, a strong association between CRELD2 expression and the recruitment of fibroblasts and the enhancement of CAF tumor‐promoting markers in cSCC. The immediate implication of this observation is that cSCCs in which CRELD2 is upregulated are likely to progress more rapidly than their counterparts in which CRELD2 levels are lower.

While the lack of gene expression data on a sufficiently large set of patient samples frustrated our attempt to demonstrate an association between CRELD2 and patient survival in cSCC, we did observe that in squamous cell carcinomas of other tissues including the lung, the expression level of CRELD2 influenced overall survival probability of patients. These data are consistent with the published observations that CRELD2 expression is highest at later stages of breast cancer and was also associated with the triple‐negative form, which has a poor prognosis.

While these data should be replicated using independent cohorts of cSCC patient specimens, the implications of our findings to the management of SCC patients are obvious. Given the relative ease with which CRELD2 levels can be quantified via immunofluorescence analysis of SCC biopsies, this could inform the treatment of individual patients on an ongoing basis. For instance, since cSCCs result from cumulative skin damage from sun exposure throughout life, and consequently most patients will exhibit multiple cSCCs over their lifetime, CRELD2 levels could be used to assist in deciding on management options for patients, with those with high CRELD2 levels monitored more frequently and assessed more closely for signs of invasion and metastasis, which have the potential to limit survival.

## 4 MATERIALS AND METHODS

### 4.1 Human cutaneous squamous cell carcinoma specimens

All procedures were performed under appropriate licenses and with the oversight of the human research ethics committee of the Central Adelaide Local Health Network and the University of South Australia Human Research Ethics Committee. Archived human cutaneous squamous cell carcinoma was obtained from SA Pathology. The study is fully compliant with all relevant ethical regulations regarding research involving human participants.

### 4.2 Histological, immunohistochemical and immunofluorescence analyses

Histology and immunohistochemistry were performed utilizing protocols we have previously established (Ibbetson et al., 2013; Samuel et al., 2009; Samuel, Lopez, et al., 2011; Samuel, Lourenco, & Olson, 2011), uniformly across all samples to ensure reproducibility of our results. All sections were of 4 μm thickness. For immunohistochemical and immunofluorescence analyses, antigen retrieval buffers, methods and antibody dilutions used are given in Table 1. Immunohistochemically stained sections on slides were imaged using a Hamamatsu Nanozoomer NDP slide scanner (Hamamatsu Photonics). Immunofluorescence images were acquired using an LSM 700 confocal microscope (Carl Zeiss) or an SP8 confocal system (Leica Microsystems, Wetzlar, Germany).

**TABLE 1:** Related to Section 4: histological, immunohistochemical and immunofluorescence analysis.

Antibody	Species	Supplier	Antigen retrieval^a^	Dilution	Incubation
					Temp (°C) [time (h)]
CRELD2	Mouse	Santa‐Cruz	10 mM Tris‐Cl; 1 mM EDTA pH 9	1:100(IF, IHC)	4[18](IF, IHC)
Cytokeratin 14	Mouse	Leica Microsystems	—	1:400(IHC)	4[18](IHC)
S100A4	Rabbit	Sigma‐Aldrich	10 mM citrate pH 6	1:250(IF, IHC)	4[18](IF, IHC)
p(Thr696)‐MYPT1	Rabbit	Merck	10 mM citrate pH 6	1:100(IF, IHC)	4[18](IF, IHC)
PDGFRB	Rabbit	Abcam	10 mM Tris‐Cl; 1 mM EDTA pH 9	1:100(IF)	4[18](IF)

^a^ All antigen retrieval was carried out in a pressure cooker at 63 kPa above atmospheric pressure, with boiling for 15 min.

*Abbreviations*: IF, immunofluorescence; IHC, immunohistochemistry.

### 4.3 Quantification of immunofluorescence signal

Fluorescence values are expressed relative to area as integrated density (i.e., fluorescence normalized to area) using ImageJ (NIH). ImageJ was used to derive integrated density values after conversion to a binary image based upon a single manually determined threshold value applied across all images as previously described (Ibbetson et al., 2013; Samuel, Lopez, et al., 2011). Results were expressed as medians, ranges and quartiles across all data sets for each histological type.

### 4.4 Statistical analyses

Violin plots show individual values (dots), medians (dashed‐colored lines) and quartiles (dashed black lines) of non‐parametric data, with *p*‐values calculated using the Kruskal–Wallis test. Data showing repeated measures is represented by point and line graphs, with points showing median values acquired from specified samples and lines linking measurements acquired in different parts of the same sample exhibiting the relevant histological characteristics. These data were analyzed using the Friedman test. In all cases, *p* < .05 was used as the significance cut‐off.

## AUTHOR CONTRIBUTIONS

Conceptualization: SJI, MSS. Data Curation: AP, EJB, MSS. Formal analysis: AP, EJB, MSS. Funding Acquisition: MSS. Investigation: AP, MSS. Methodology: AP, STB, MSS. Project administration: MSS. Resources: SJI, MSS. Software: Not applicable. Supervision: STB, MSS. Validation: AP, SJI, MSS. Writing – Original Draft Preparation: MSS. Writing – Review & Editing: AP, STB, SJI, MSS.

## CONFLICT OF INTEREST STATEMENT

None.

## Data Availability

The data that support the findings of this study are available from the corresponding author upon reasonable request.
